# Fetal Pain: Neurobiology, Clinical Practice, and Compassionate Care—A Narrative Review

**DOI:** 10.5041/RMMJ.10583

**Published:** 2026-10-26

**Authors:** Omri Dominsky, Ido Solt

**Affiliations:** 1 Department of Obstetrics and Gynecology, Lis Hospital for Women’s Health, Tel Aviv Sourasky University Medical Center (Ichilov), Tel Aviv, Israel (affiliated to the Gray Faculty of Medical and Health Sciences, Tel Aviv University, Tel Aviv, Israel); 2 Department of Obstetrics and Gynecology, Rambam Health Care Campus, Haifa, Israel; 3 The Ruth and Bruce Rappaport Faculty of Medicine, Technion–Israel Institute of Technology, Haifa, Israel

**Keywords:** Compassionate care, fetal analgesia, fetal pain, fetal surgery, nociception

## Abstract

**Background:**

Whether a fetus can experience pain remains scientifically and clinically contested, particularly as invasive fetal procedures become more common.

**Objectives:**

To review fetal nociceptive development, the evidence and uncertainties relevant to pain perception, and the clinical rationale for fetal analgesia during invasive procedures.

**Methods:**

We performed a targeted narrative search of PubMed/MEDLINE, Google Scholar, the Cochrane Library, reference lists, and professional society websites. The final synthesis comprised 34 core sources.

**Results:**

Functional nociceptive and neuroendocrine responses to noxious stimuli are documented from the mid-second trimester and can be attenuated by fetal opioids. Early thalamic and subplate circuitry may challenge the claim that mature cortical architecture is required for all pain-related processing, but this circuitry has not been shown to be sufficient for conscious pain. Continuous endogenous fetal sedation is not supported by behavioral evidence, although the fetal neurochemical environment may modulate arousal and sensory processing. Maternal anesthesia, especially regional anesthesia, may not provide reliable fetal analgesic exposure. Professional guidance differs on the gestational age at which pain perception becomes possible.

**Conclusions:**

The clinical case for fetal analgesia should be separated from the unresolved question of conscious pain. Potential rationales include preventing possible suffering, attenuating documented physiological stress responses, and facilitating procedural stability. Decisions should therefore be based on a proportionate, procedure-specific maternal-fetal risk-benefit assessment rather than a universal precautionary default.

## INTRODUCTION

Few topics in perinatal medicine demand such careful consideration of scientific complexity, clinical urgency, and ethics as fetal pain. The question of whether a fetus can experience pain matters not only philosophically but practically: with recent advances in fetal surgery, approximately 1,500 fetal surgical procedures are performed annually in the United States alone, and many thousands more intrauterine interventions, including transfusions, fetoscopic procedures, and cases of selective feticide, are carried out worldwide each year.^1^ The clinicians performing these procedures must decide whether the fetus needs analgesic protection, and if so, how to provide it.

The history of this debate is instructive. Until the final decades of the twentieth century, the scientific community denied not only fetal pain but even neonatal pain. As late as the 1980s, many clinicians performed invasive procedures on newborns without adequate analgesia, citing concerns about the risks of opiates rather than evidence of insensitivity. Anand and Hickey’s landmark 1987 paper helped establish that neonates mount substantial physiological stress responses to noxious procedures and that adequate anesthesia was associated with improved immediate clinical outcomes, including lower morbidity and mortality.^2^ The extension of that work to the fetus followed soon afterward, and the debate has been evolving ever since.

This review follows the arc of that debate: from the first physiological evidence of fetal nociception, through neuroanatomical and pharmacological objections, to current clinical practice. Throughout, we distinguish three questions that are related but not equivalent: whether the fetus detects and responds to noxious stimuli, whether those responses imply a subjective pain experience, and whether fetal analgesia is clinically justified. We also examine what remains genuinely uncertain, because intellectual honesty about those uncertainties is itself a form of compassionate medicine. This review addresses analgesia in the context of invasive clinical fetal procedures. It does not constitute a position statement on termination of pregnancy; selective feticide is mentioned only in its clinical context as an invasive intrauterine intervention.

## METHODOLOGY

This narrative review was informed by a targeted literature search. Searches were performed in PubMed/MEDLINE, Google Scholar, the Cochrane Library, and professional society websites, including the American College of Obstetricians and Gynecologists (ACOG), Society for Maternal-Fetal Medicine (SMFM), the Royal College of Obstetricians and Gynaecologists (RCOG), and the International Federation of Gynecology and Obstetrics. Search terms included combinations of “fetal pain,” “fetal nociception,” “fetal stress response,” “fetal consciousness,” “pain perception,” “thalamocortical connections,” “cortical development,” “fetal anesthesia,” “fetal analgesia,” “invasive fetal procedures,” “fetal surgery,” “placental transfer,” “opioids,” “remifentanil,” “fentanyl,” “maternal-fetal anesthesia,” and “ethical considerations.” Reference lists of key publications were also reviewed. Sources were selected when they addressed fetal neurodevelopment, nociceptive pathways, physiological responses to invasive procedures, clinical fetal analgesia/anesthesia, pharmacology, or relevant professional and ethical guidance. Sources focused primarily on political or legal debate, non-human models without clear translational relevance, duplicate material, or topics not directly relevant to invasive fetal procedures were not included as core references. The final narrative synthesis comprised 34 core sources.

Pain-related terminology is used with caution throughout this review. *Nociception* refers to the detection and neural transmission of noxious stimuli and may occur without conscious awareness. *Physiological stress responses*, including hormonal, hemodynamic, or autonomic changes, indicate activation of fetal stress pathways but do not by themselves prove pain perception. *Pain perception* implies that noxious input is processed as an unpleasant sensory-affective experience, while *conscious experience* refers to the broader capacity for subjective awareness. Because none of these phenomena can be directly reported by the fetus, clinical interpretation must rely on indirect evidence while avoiding the assumption that reflex movement, immobility, or stress responses are equivalent to conscious pain.

The scope of this review is restricted to invasive fetal procedures and the clinical question of whether, when, and how fetal analgesia should be considered during interventions that may involve direct fetal tissue injury. The paper does not present an ethical, legal, or political position on abortion or termination of pregnancy. When termination-related procedures are mentioned, they are discussed only as examples of invasive intrauterine interventions in which questions of fetal nociception, physiological stress responses, analgesia, and maternal–fetal risk may arise. This distinction is important: the purpose of the review is to inform clinical pain-management considerations in fetal medicine, not to adjudicate broader moral or political debates.

## THE FIRST EVIDENCE: FETAL STRESS RESPONSES TO INVASIVE STIMULI

The earliest direct evidence that fetuses respond to potentially painful stimuli came from Fisk, Giannakoulopoulos, and colleagues at King’s College London in the 1990s. Their elegant experimental design compared fetal hormonal responses to needling of an innervated site, the intrahepatic umbilical vein, with responses to needling at the non-innervated placental cord insertion. Fetuses needled at the innervated site showed significant elevations in plasma cortisol, noradrenaline, and beta-endorphin within minutes; those needled at the non-innervated site showed no such response.^3^,^4^ This internal control established that the hormonal response was nociception-specific rather than a non-specific procedural effect.

In a subsequent randomized controlled trial, the same group demonstrated that direct fetal administration of fentanyl before intrahepatic vein needling significantly attenuated both hormonal and hemodynamic stress responses.^4^ Not only does the fetus mount a measurable physiological stress response to noxious stimuli, but that response is pharmacologically suppressible by opioid analgesia. Whether this reflects conscious pain experience or subcortical nociceptive processing remains debated; what it establishes unambiguously is that the fetus detects, transmits, and responds to noxious stimuli through functional neuroendocrine pathways from at least the mid-second trimester.

These studies established several points that now form the empirical foundation of the field. The fetal hypothalamic–pituitary–adrenal axis is functionally responsive to noxious stimuli from at least 18–20 weeks of gestation, independently of maternal hormone levels.^5^ The response is site-specific, dose-dependent, and opioid-reversible. Critically, it is present long before the gestational ages at which the cortical necessity arguments would permit any pain experience at all.

## THE OBJECTIONS: CORTICAL IMMATURITY AND THE PAIN MATRIX

The physiological evidence was compelling, but critics raised a fundamental neuroanatomical objection: to feel pain in any meaningful sense, the argument went, a developed cerebral cortex is necessary. Lee and colleagues, in an influential 2005 review in *JAMA*, argued that the capacity for conscious pain perception could arise only after thalamocortical pathways begin to function, which they placed at 29–30 weeks of gestational age.^6^ Derbyshire went further, suggesting that a response worthy of the name pain required approximately 12 months of postnatal brain development.^7^ Under this framework, fetal reactions to noxious stimuli before the late third trimester were purely spinal reflex responses, devoid of any experiential dimension.

A related debate concerned the activation of the so-called pain matrix, a distributed brain network activated by painful stimuli that has also been observed in fetuses and premature neonates. Some authors argued that activation of this network was sufficient evidence that the fetus was experiencing pain.^8^ Others countered that pain matrix activation is nonspecific and can occur in response to stimuli that are not experienced as painful, making it an unreliable indicator of conscious pain experience.^9^

These objections have prompted greater attention to structures other than the mature cortical plate. The cortical subplate, a transient but functionally active neuronal layer that receives thalamic input before mature thalamocortical connections enter the cortical plate, is present and active from approximately 20 weeks of gestation.^10^,^11^ The thalamus is also substantially developed earlier in gestation.^12^ Observations in neonates with severe cortical malformations have been interpreted as suggesting that subcortical structures may support rudimentary affective or arousal-related states.^13^ However, demonstrating that mature cortical architecture may not be necessary for all nociceptive processing is not equivalent to demonstrating that thalamic or subplate circuitry is sufficient for conscious pain. These developmental findings therefore weaken a simple cortex-or-no-pain dichotomy, but they do not establish fetal subjective experience.

The question of whether conscious experience is possible without a fully functioning cortex connects to a broader debate about consciousness itself. Lagercrantz and others have proposed that rudimentary consciousness may emerge gradually from approximately 24 weeks of gestation, with increasing integration thereafter.^14^,^15^ Fetal magnetoencephalography has demonstrated brain responses consistent with higher-order processing by approximately 27 weeks.^16^ Studies of sleep, anesthesia, and disorders of consciousness also show that noxious input can influence neural activity and behavior in states of reduced awareness.^17^,^18^ These observations are relevant to the debate, but they do not provide a direct test of fetal pain experience.

## OVERCOMING THE SEDATION HYPOTHESIS

A second major argument against fetal pain held that even if nociceptive pathways were present, the fetus is maintained in a continuous state of sleeplike unconsciousness by endogenous neuroinhibitors, including adenosine, progesterone, and prostaglandins, present in fetal blood. The RCOG’s 2010 report endorsed this view, concluding that the fetus never experiences a state of true wakefulness *in utero* and is kept, by the presence of its chemical environment, in a continuous sleep-like unconsciousness or sedation.^6^,^19^ Under this double framework, cortical immaturity ruled out pain before 24 weeks, and chemical sedation made it unlikely even after.

This argument has been systematically challenged. Bellieni and colleagues demonstrated that fetuses are awake approximately 9%–21% of their time in the final weeks of gestation, exhibit distinct behavioral states including quiet wakefulness (3F) and active wakefulness (4F), and can be aroused by external stimuli including vibroacoustic stimulation and maternal vocalization.^20^,^21^ These behavioral observations do not support the claim that the fetus is continuously unconscious.

The endogenous sedation hypothesis also requires a more nuanced interpretation. Comparisons between fetal and maternal blood concentrations of neuroinhibitory substances are not sufficient to infer equivalent functional effects, because developmental responses depend on receptor expression, network organization, metabolism, and interacting neurochemical systems. The stronger point is conceptual: sedation, sleep-like quiescence, and analgesia are not synonymous. Behavioral evidence argues against continuous fetal unconsciousness, but the intrauterine neurochemical environment may still modulate arousal and nociceptive processing. Accordingly, rejecting a state of continuous sedation does not itself demonstrate conscious pain.

In its updated 2022 evidence review, the RCOG no longer relied on continuous endogenous sedation as a central argument and concluded that pain perception before 28 weeks’ gestation remains unlikely.^22^ Bellieni subsequently argued that, although the 2022 review improved on the 2010 document, further revision was still warranted.^23^ Comparisons with gestational-age-matched premature neonates are clinically provocative because analgesic care is standard in neonatology, but such comparisons do not by themselves resolve whether the intrauterine environment or developmental state supports the same subjective experience. The disagreement therefore concerns interpretation of developmental evidence rather than the existence of fetal nociceptive and stress responses.

## EVOLVING EVIDENCE ON FETAL NOCICEPTION AND PAIN

Recent literature increasingly separates evidence for nociception from the more difficult inference of conscious pain. Some authors have argued that a pain-related experience is biologically plausible from approximately 20–22 weeks of gestation.^24^ In this review, “biologically plausible” means that available neurodevelopmental evidence does not exclude a minimal affective experience at that stage; it does not mean that such experience has been demonstrated, that a specific gestational threshold has been validated, or that the relevant circuitry has been shown to be sufficient. Derbyshire and Bockmann’s 2020 reconsideration is historically important because it illustrates how one influential interpretation changed, but an author’s change of view is not evidence in itself.^8^ The evidentiary question remains what the anatomical, physiological, behavioral, and clinical data can support.

The Fetal-7 scale, a recently validated fetal pain-related behavioral scoring system, provides a practical clinical tool consistent with this evolution. Using three-dimensional ultrasound recordings of fetuses undergoing preoperative anesthetic injections, predominantly in the third trimester, the scale documents complex facial responses to acute noxious stimulation.^25^ Such behavioral patterns may contribute to clinical assessment but, like hormonal or autonomic responses, cannot by themselves establish a subjective pain experience. Its applicability to earlier gestational ages also requires further study. Table 1 summarizes key neurodevelopmental milestones relevant to fetal nociceptive and pain-related processing.^10^,^12^,^26^

**Table 1:** Key Milestones Relevant to Fetal Nociceptive Development and Pain-related Processing.

Gestational Age	Developmental Milestone	Clinical Interpretation
6–12 wk	Cutaneous nerve terminals begin to form; substance P is detectable from approximately 10–12 weeks; myelination begins later	Early nociceptive neurochemistry is present, but this does not imply pain perception
13–16	wk Synapses are established in the dorsal horn; ascending spinothalamic pathways are reported from approximately 15 weeks	Basic ascending nociceptive pathways are developing
17–20 wk	Thalamic and subplate structures relevant to sensory processing are developing; endogenous opioidergic systems are present	Subcortical and early thalamic-subplate processing may contribute to nociceptive transmission, but conscious pain perception remains uncertain
18–20 wk	Fetal hormonal and hemodynamic stress responses to invasive noxious stimuli have been documented and can be attenuated by direct fetal opioid administration	Strong evidence of noxious-stimulus-evoked physiological stress responses; supports consideration of fetal analgesia during selected invasive procedures
23–24 wk	Thalamocortical connections begin to enter the cortical plate; cortical activity becomes increasingly detectable	Important transition toward cortical sensory processing; not a definitive threshold for conscious pain
26–30 wk	Somatosensory evoked potentials, fetal MEG responses, and wake-state EEG patterns become increasingly evident across this period	Progressive cortical integration of sensory and nociceptive signals; this may increase the biological plausibility of pain experience, but no validated gestational threshold for conscious pain is established

EEG, electroencephalography; MEG, magnetoencephalography.

## CLINICAL IMPLICATIONS: ANALGESIA FOR FETAL PROCEDURES

The evolution of scientific understanding has translated, unevenly but progressively, into clinical practice. Fetuses undergoing open surgical repair of myelomeningocele, resection of sacrococcygeal teratomas, *in utero* treatment of large lung masses, or *ex utero* intrapartum treatment procedures typically receive general anesthesia via maternal inhalational agents supplemented by direct fetal intramuscular administration of opioids and muscle relaxants.^27^ Intrauterine transfusion, fetoscopic laser coagulation, and procedures involving selective feticide are increasingly accompanied by direct fetal analgesia at centers that have formally adopted such protocols.

A pharmacological reality that deserves wider clinical awareness is that maternal anesthesia does not necessarily provide reliable fetal analgesic exposure. This is particularly relevant when maternal anesthesia is regional rather than general. Bellieni’s review of placental transfer data reports variable fetal exposure to commonly used agents, including substantially lower fetal concentrations of propofol and minimal fetal exposure after maternal intrathecal morphine.^12^ Thus, adequate maternal anesthesia should not automatically be assumed to provide equivalent fetal analgesia; the required strategy depends on the procedure, route, drug, timing, and intended fetal effect (Table 2).

**Table 2:** Placental Transfer and Clinical Considerations for Analgesic Agents Used in Fetal Procedures.

Agent	Placental Transfer/Fetal Exposure	Route/Context	Clinical Consideration
Fentanyl	Crosses the placenta; fetal exposure depends on maternal dose, timing, and route. Direct fetal administration provides more predictable fetal exposure.	Direct fetal intramuscular or intravascular administration; maternal intravenous fentanyl may also contribute to fetal exposure, depending on dose and timing.	Direct fetal administration provides the most predictable opioid exposure. Published practice commonly uses approximately 10-20 micrograms/kg estimated fetal weight.^28^ Maternal fentanyl alone should not be assumed to provide equivalent fetal analgesia.
Remifentanil	Rapid placental transfer with rapid metabolism by maternal and fetal esterases.	Maternal IV infusion, especially when procedures are performed under regional anesthesia.	May reduce fetal movement and provide fetal opioid exposure in selected settings but requires careful maternal respiratory and hemodynamic monitoring. It should not be assumed to provide equivalent coverage to direct fetal opioid administration.
Morphine	Placental transfer occurs after systemic maternal administration, but fetal levels after neuraxial maternal administration may be low or negligible.	Maternal systemic or neuraxial routes.	Maternal neuraxial morphine should not be assumed to provide adequate fetal analgesia. Clinical utility for fetal analgesia depends strongly on route and timing.
Sevoflurane/volatile anesthetic agents	Cross the placenta to variable degrees during maternal general anesthesia.	Maternal inhalational general anesthesia, especially open fetal surgery or EXIT procedures.	May contribute to fetal anesthesia and uterine relaxation during maternal general anesthesia. Safety should be discussed in terms of dose, duration, and indication; avoid stating simply that exposure is “safe if <3 hours.”
Propofol	Crosses the placenta, but fetal concentrations may be substantially lower than maternal concentrations depending on timing and dose.	Maternal IV anesthesia or sedation.	Should not be assumed to provide reliable fetal analgesia as a sole agent during invasive fetal procedures.
Vecuronium/pancuronium	Limited placental transfer after maternal administration; direct fetal administration produces fetal paralysis.	Direct fetal IM administration.	Provides fetal immobilization only. It is not analgesic and should be combined with an analgesic agent when fetal pain or nociceptive stress is a concern.

EXIT, *ex utero* intrapartum treatment; IM, intramuscular; IV, intravenous.

Fentanyl, administered intramuscularly at approximately 10–20 micrograms per kilogram of estimated fetal weight, is the most widely adopted agent for direct fetal analgesia.^28^ Neuromuscular blocking agents such as vecuronium or pancuronium are often added for procedural immobilization, but a critical distinction must be maintained: immobilization is not analgesia, and the absence of fetal movement during a procedure cannot be interpreted as the absence of pain. Remifentanil, given its high placental permeability and rapid placental metabolism, offers a useful alternative in procedures performed under maternal regional anesthesia.^12^

An important cautionary note concerns the potential neurodevelopmental effects of repeated or prolonged anesthetic exposure. The United States Food and Drug Administration has warned that repeated or lengthy use of general anesthetic and sedation drugs in children younger than 3 years or in pregnant women during the third trimester may affect brain development.^1^ The warning was based largely on animal data and limited human evidence, and its relevance to brief, single-dose fetal analgesia, particularly in the second trimester, remains uncertain. Prospective data specific to fetal interventions are needed.

Direct fetal analgesic administration carries procedural and pharmacological risks that must be weighed against its potential benefit. At the procedural level, intramuscular or intravascular fetal injection under ultrasound guidance carries a small but real risk of inadvertent vessel puncture, fetal bradycardia, and in rare cases fetal loss, with rates dependent on operator experience and gestational age.^27^ At the pharmacological level, opioid administration may cause transient fetal heart rate changes and neonatal respiratory depression if delivery occurs shortly after administration; neuromuscular blocking agents carry a risk of prolonged fetal immobility if dosing is imprecise. At the maternal level, additional drug administration increases procedural complexity and duration. These risks must be weighed against the potential benefits of fetal analgesia and reinforce the importance of procedure-specific risk-benefit assessment rather than a uniform approach across all fetal interventions.

Professional guidance illustrates the distinction between the disputed question of conscious pain and the clinical use of fetal analgesia. Ethical guidance from the International Federation of Gynecology and Obstetrics addresses pain relief for fetuses and neonates but does not establish a specific neurodevelopmental threshold for fetal pain.^29^ The SMFM Consult Series #59, endorsed by ACOG and supported by RCOG, states that fetal analgesia in maternal-fetal procedures is used primarily to blunt autonomic responses and minimize fetal movement. Although the document considers conscious pain unlikely at the gestational ages when many procedures are performed, it suggests fetal opioid analgesia during invasive fetal surgery to attenuate acute autonomic responses, reduce potential consequences of nociception and physiological stress, and facilitate safe performance of the procedure.^30^ The current ACOG statement similarly states that a fetus does not have the capacity to experience pain until after at least 24–25 weeks of gestation and explicitly notes that anesthesia or analgesia during fetal surgery may be appropriate for reasons unrelated to pain perception. 31 The 2022 RCOG review concludes that pain perception before 28 weeks remains unlikely.^22^

A more cautious interpretation of the evidence has long been advanced by RCOG^22^ and Lee et al.,^6^ who emphasize that withdrawal movements, facial activity, neuroendocrine stress responses, and other nociceptive phenomena should not be equated with conscious pain. Their position underscores an important evidentiary asymmetry: observable responses establish that noxious information is detected and processed, but the subjective quality of that processing remains inferred. Accordingly, the present review does not argue for a categorical gestational threshold or a universal mandate for fetal analgesia.

## DEFINING PAIN AND THE LIMITS OF CURRENT TERMINOLOGY

The International Association for the Study of Pain revised its definition of pain in 2020, describing it as “an unpleasant sensory and emotional experience associated with, or resembling that associated with, actual or potential tissue damage,” and clarifying that “verbal description is only one of several behaviors to express pain; inability to communicate does not negate the possibility that a human or a nonhuman animal experiences pain.”^32^

Canepa and colleagues have argued that even this revised definition remains inadequate for neonates and fetuses, and that clinical medicine must rely on behavioral, hormonal, and physiological indicators as proxies for pain experience in these populations.^32^ This is not a counsel of despair but a reminder that clinical judgment must fill the gaps that definitions cannot.

## SEPARATING CLINICAL RATIONALES FROM THE CONSCIOUSNESS QUESTION

The clinical decision to administer fetal analgesia should be separated from the unresolved question of subjective experience. At least three rationales can be distinguished: (1) preventing possible conscious suffering; (2) attenuating nociceptive, autonomic, and neuroendocrine stress responses; and (3) facilitating the procedure through physiological stability and/or immobility. Only the first depends directly on the disputed inference of fetal consciousness. The latter two may justify analgesia even under a skeptical view of fetal pain, as reflected in current SMFM guidance.^30^

This distinction also clarifies the role of precaution. A precautionary principle cannot identify a single protective action when both intervention and non-intervention carry risks. Birch’s proportional framework argues that precautions should be calibrated to the magnitude and plausibility of harm,^33^ while Derbyshire emphasizes that where analgesia improves physiological or procedural outcomes it can be justified independently of unresolved claims about subjective pain.^34^ For fetal procedures, the relevant assessment should therefore consider gestational age, invasiveness, expected stress response, feasibility and risks of direct fetal dosing, maternal anesthetic exposure, procedural complexity, and the likelihood of imminent delivery.

The principle of compassion in medicine remains relevant but should not be understood as dictating a single precautionary default. Compassion requires taking seriously both the possibility of fetal suffering and the possibility of iatrogenic maternal or fetal harm. When analgesia can attenuate established stress responses or facilitate safe fetal surgery with an acceptable risk profile, its use may be justified independently of proof of conscious pain. Conversely, when incremental fetal benefit is uncertain and drug or procedural risk is appreciable, a different strategy may be more protective. Compassion is therefore best operationalized through proportional risk-benefit assessment, transparent communication, and procedure-specific judgment.

## CONCLUSIONS: WHAT REMAINS TO BE CLARIFIED

The available evidence supports a differentiated conclusion. Functional nociceptive pathways and measurable neuroendocrine and hemodynamic stress responses are present by the mid-second trimester. The claim that the fetus is continuously sedated is not supported by behavioral evidence, and mature cortical architecture may not be the only substrate relevant to pain-related processing. However, current data do not establish when subjective pain experience begins, and early thalamic or subplate function is not in itself evidence that those circuits are sufficient for conscious pain. Fetal pain before later gestational stages therefore remains an area of genuine epistemic uncertainty rather than a demonstrated fact.

Important questions remain open. We do not know whether the emergence of pain experience, if present prenatally, occurs at a sharp threshold or gradually, nor do we know its quality or intensity. Most evidence is indirect and derives from anatomy, physiology, behavior, and responses to analgesia rather than from any direct measure of subjective experience.^6^ Terminology is also difficult: standard definitions of pain were developed for communicative patients, and their application to fetuses requires careful conceptual adaptation.^32^ Finally, fetal analgesic pharmacology remains incompletely defined. Placental transfer varies by drug and route, direct fetal administration has procedural risks, and the relevance of concerns about prolonged anesthetic exposure to brief fetal interventions remains uncertain.^1^

These uncertainties do not preclude clinical action, but they change its justification. Decisions about fetal analgesia should be grounded in a procedure-specific balance of maternal and fetal risks and benefits. Prevention of possible conscious suffering is one consideration, but attenuation of documented physiological stress responses and facilitation of a safe, stable procedure provide independent clinical rationales. Compassionate care therefore means neither automatic treatment nor automatic withholding; it means proportional protection, honest communication about uncertainty, and reassessment as the evidence evolves.

### Take-home Messages

- Functional fetal nociceptive pathways and hormonal/hemodynamic responses to noxious invasive stimuli are documented from the mid-second trimester and can be attenuated by opioid analgesia; these findings establish nociception and physiological stress, not conscious pain by themselves.
- The clinical rationale for fetal analgesia can be independent of the consciousness debate: potential benefits include attenuation of autonomic and neuroendocrine stress responses and facilitation of procedural stability, while maternal and fetal drug/procedural risks must also be considered.
- No gestational age has been definitively established as marking the onset of fetal pain perception. Clinical decisions should therefore use a proportionate, procedure-specific maternal-fetal risk-benefit assessment rather than a universal precautionary default.

## Abbreviations

ACOG: American College of Obstetricians and Gynecologists
RCOG: Royal College of Obstetricians and Gynaecologists
SMFM: Society for Maternal-Fetal Medicine
